# Blockchain Technology for Trustworthy Operations in the Management of Strategic Grain Reserves

**DOI:** 10.3390/foods10102323

**Published:** 2021-09-29

**Authors:** Adnan Iftekhar, Xiaohui Cui, Yiping Yang

**Affiliations:** Key Laboratory of Aerospace Information Security and Trusted Computing, Ministry of Education, School of Cyber Science and Engineering, Wuhan University, Wuhan 430072, China; adnan@whu.edu.cn (A.I.); yangyiping@whu.edu.cn (Y.Y.)

**Keywords:** food security, strategic grain reserve, blockchain technology, hyperledger fabric

## Abstract

Food is a daily requirement for everyone, while production patterns are seasonal. Producing sufficient nutrients is becoming more difficult because water and soil resources are already stressed and are becoming increasingly strained by climate change. Improving food security requires expertise in various areas, including sophisticated climate models, genetics research, market and household behaviour modelling, political shock modelling, and comprehensive environmental research. Additionally, governments stockpile grains to enhance national food security. These reserves should engage in markets only according to clear and transparent regulations and within defined price ranges to facilitate market functioning. It increases the demand for better technology in public administration to boost the management and distribution capacity while concentrating on improved controls and transparent governance systems. Blockchain technology emerges as a promising technology to enhance visibility, transparency, and data integrity with an immutable distributed ledger to increase trust in the parties’ business transactions. This paper discusses blockchain technology and its potential role in strategic grain reserve management.

## 1. Introduction

**Figure 1:**
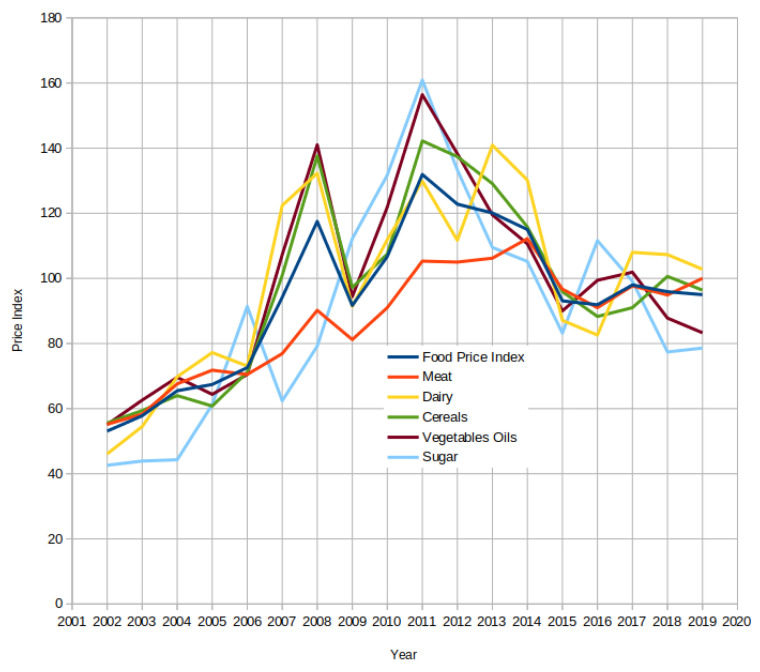
The price surge of food commodities during 2007–2008 and 2011–2012.

The strategic grain reserves are stockpiles of food in the form of grains. In developing countries, strategic grain reserves are an integral part of agricultural policies to stabilize food prices [1]. These grain reserves sources serve food-insecure households at subsidized prices during some shortage in the market and supply food to the areas struck by natural or human-generated disasters. The rapid increased in the prices of many commodities during 2007–2008 shocked the governments and consumers in many parts of the world [2]. Figure 1 shows a significant surge in food prices during 2007–2008 and 2011–2012 based on FAO data. According to the Food and Agriculture Organization of the United Nations (FAO), 35 countries with strategic grain reserves, including Cambodia, Cameroon, China, Ethiopia, India, Kenya, Nigeria, Pakistan, and Senegal, used their stocks to provide food at subsidized prices to their people. These countries responded more quickly and economically than those with limited or no reserves [3]. The 2009 G-8 and G20 summits and the FAO World Food Security Summit acknowledged the strategic grain reserves’ significance [4]. Regional organizations such as the Association of Southeast Asian Nations (ASEAN) and the Economic Community of West African States (ECOWAS) in West Africa also stated their intention to establish regional strategic grain reserves. It will help these governments manage food surplus and food shortages within the same region in a short period.

Many governments across the globe have either abandoned or significantly reduced their grain reserve initiatives during the last three decades. The food crisis exposed the weakness of depending only on the market to handle growing uncertainty and fluctuating pricing in agricultural markets. Food security is contingent upon a healthy balance of commerce and self-sufficiency. In developing countries, reserve creates a guaranteed market that encourages investment in agricultural products and distribution systems. Governments procure significant crops such as wheat, corn, and rice to encourage farmers to increase agricultural production. At the same time, the export of excessive output is a significant source of foreign exchange for countries [5]. However, the larger companies engaged in the agricultural and food businesses have superior access to resources and market knowledge than the governments. These companies impact food policy in their favour by using their lobby in the governments. The grain reserves need to be transparent and well-governed.

Grain reserves cost money. The management and release of public stocks are often coupled with subsidised sales of food. There is a lack of effective coordination, auditing, and sharing of data across departments and offices in developing countries. Moreover, most of the procedure is manual and paper-based. No department takes any responsibility for missing or corruption in records or data in the case of any incident or inquiry. The most significant challenge for the good governance of grain reserves is to ensure linked data throughout the supply chain. The existing IT solutions reduced some of these challenges. However, there is still a lot of undetected frauds and transparency levels. Blockchain technology is a novel digital method to guaranteeing data integrity and preventing tampering. It is a peer-to-peer distributed ledger system that prevents single point failure by providing fault-tolerance, immutability, trust, transparency, and full traceability of the stored transactions. A potential solution to alleviate the above issues and concerns is the use of blockchain technology in the management of the strategic grain reserves. The system attempts to ensure data sharing and integrity by storing it in a single shared ledger, thus meeting the transparency criteria necessary to avoid corruption and mismanagement.

The rest of this paper is arranged as follows. In Section 3, we describe the Blockchain Technology without involving technical details to make it understandable for government officials and policy makers. In Section 5, we discuss the generic architecture of the proposed solution. In Section 6, we present the potential qualitative analysis of the proposed solution followed by the conclusion of the article in Section 7.

## 2. Challenges in Emerging Economies

In developing countries, government purchases of important crops are a kind of compensation for farmers. Fraud and mismanagement in the purchase and distribution system also directly impact farmers and their ability to earn a living and producing food. Farmers are forced to sell their produce at a lower price than the government-set rates because of the malpractices of intermediaries and private sector business organizations [6]. The intermediaries, for the most part, charge a large commission from the farmers to facilitate sales of theirs crops. Farmers in emerging countries suffer directly due to the government’s inability to regulate the grain markets. Farmers are also compelled to sell their production at cheap rates to intermediaries due to the poor management of the government’s food and agriculture departments. These intermediaries create fake shortages to drive up prices and then resell the product at inflated rates back to the market. It has been reported that thousands of individuals who work in the agricultural industry committed suicide in India due to these issues [7]. The Indian farmers’ protest is an ongoing demonstration against the 2020 Indian agricultural reforms, as they describe these reforms as putting the farmers at the mercy of multinational corporations [8]. Some of the most frequent issues are listed below.

- **Corruption & Bribe**
  Bribery is a common form of procurement fraud that occurs on a large scale. After receiving the money for crops, the farmer pays this bribe to the administrative personnel on the other side. It may range anywhere from 5 percent to 10 percent. The majority engaged in this operation are intermediaries and brokers, who also get a portion of the proceeds.
- **Influence Usage**
  Over time, lower quality goods are bought at exorbitant costs. The vendor subsequently made a payment to the administrative personnel in the amount of the percentage. This strategy was used by the influenced brokers and intermediates, including the landlords engaged in politics and government.
- **Ghost Billing**
  The billing will be in the form of overweight. It is also found mud and bricks into the crop’s bags instead of the crop for making overweight billing and later removed before storage. The excess payment for the overweight later goes to the administrative staff.
- **Conflict of Interest**
  Many of the individuals who hold positions of power in the government are industrialists. They set the pricing of crops according to their interests and encourage corruption to purchase products from farmers at lower prices than those set by the government.

**Figure 2:**
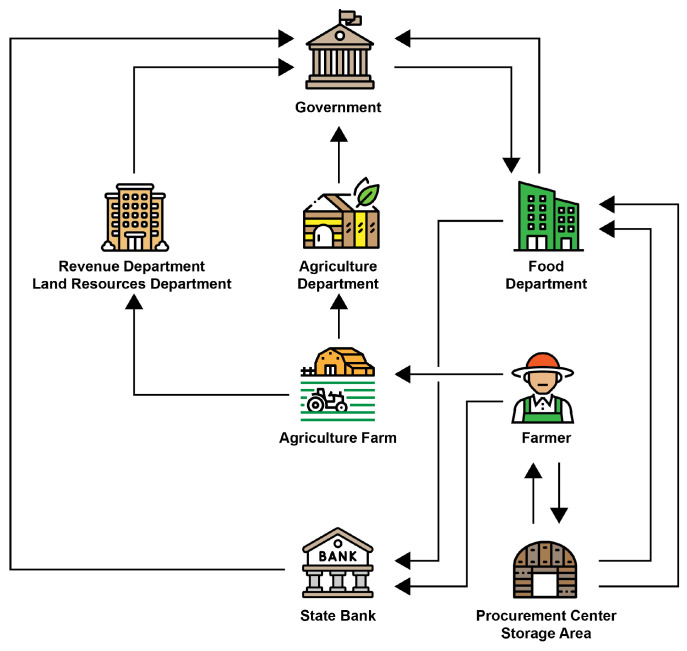
A traditional system of reserve grain management in the subcontinent region.

An abstract model, loosely based on the traditional procurement system in the subcontinent, is illustrated in Figure 2. The governments have land resources or a revenue department that holds barren or fertile agricultural land, commercial, and residential lands. The agricultural department conducts the survey twice a year to record the farmer, the crop, and its expected yield. The government makes a procurement and export policy based on these reports. This policy then executes through the food departments. The food department creates a consortium with state banks and other regional organizations to purchase directly from the farmers. The farmer brings their production to the designated purchase centers, confirming the farmer’s purchase receipt’s quality assurance and issues. The purchase center sends a payment scroll to the bank to make payments to the farmer through the state-owned banks. The food department is also responsible for releasing these stocks to the flour mills and public through designated centers.

There is no proper coordination, auditing, and sharing of the data between the departments and the offices throughout the regions and country. It also causes the following problems to design an effective big data analytics tool.

- The lack of data accuracy
- The outdated data and low latency of data transferring
- Security and Privacy of the data

**Figure 3:**
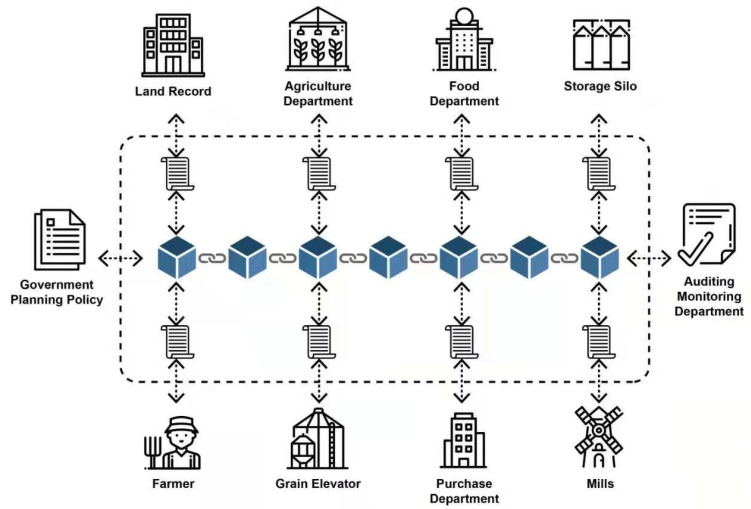
Logical overview of the proposed blockchain-based system.

To eliminate these issues, we proposed adopting blockchain technology due to several advantages, such as all the nodes in blockchain are connected to a single logical channel. Its records and shared all the time-stamped and cryptographically secured transactions with all the channel members in almost real-time without the requirement of any additional auditing and involvement of third party department (Figure 3).

**Figure 4:**
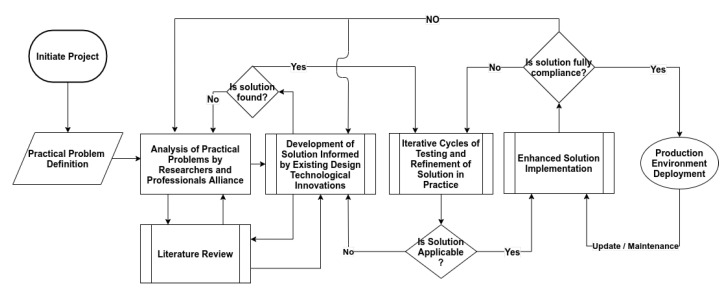
Research Methodology.

We already published several articles on blockchain applications and deployed blockchain technology in the food safety area with an alliance of food producers, and distributors in China’s Hubei province [9,10,11,12,13,14]. The purpose of our work is more about the horizontal applications of blockchain technology in social computing in developing countries. It is more about shaping a food system for tomorrow’s digital world rather than making a blockchain technological stack but where it is needed. We developed a design-based research approach with the concept of mindful use of information technology [9]. The mindful use of technology emphasizes employing the most efficient and cost-effective technological elements to solve problems. The design-oriented approach focuses on analyzing real-world practical issues via researchers and professionals to create a solution that uses current design principles, and technology advances [15]. These solutions are subsequently developed and improved with the research and development needed to address a specified issue in the production environment. Figure 4 shows the whole process of our proposed technique based on a design-based research approach.

## 3. Blockchain Technology

In 2013, the German government decided the start of the Fourth Industrial Revolution (Industry 4.0) [16]. Traditional manufacturing and industrial processes are being continuously automated as part of the Fourth Industrial Revolution, which is being driven by contemporary smart technologies. In today’s world, sensors are the building blocks of technologies such as intelligent homes and cities, smart grids, intelligent health systems, and wearable technology [17]. The Internet of Things (IoT) achieved considerable momentum in the automation industry. As the number of IoT devices grows, the volume of data generated by them will also grow. Managing these rapidly expanding IoT devices and enormous data efficiently to be available to all authorized users without compromising its integrity will become essential in the near future. On the other side, many information security incidents have been recorded, increasing the requirement for countermeasures. While safeguards against hostile third parties have been commonplace until now, operators and parties have seen an increase in demand for data falsification detection and blocking. Blockchain technology is well-known for its privacy, immutability, and decentralized nature.

Since the introduction of cryptocurrency Bitcoin [18] in response to the 2007–2008 global financial crises, Blockchain technology has become a point of interest among researchers and developers. A blockchain is a decentralized, trustless verification system based on cryptography, peer-to-peer distributed ledger, forged by consensus, and combined with a system for smart contracts and other assistive technologies. Each party participating in this network has an exact copy of the ledger throughout the network. It also allows creating a single instantaneous source of truth. These transactions are secure and verifiable in a transparent way. The main advantage of blockchain over the existing technologies is that it enables the two parties to make transactions over the Internet securely without any intermediary party’s interference [19].

A blockchain consist of blocks of information. The block consists of data, the hash of the previous block, and the current block’s hash. The data depends on the type of the blockchain. In the bitcoin blockchain, the data consists of the transactions and have senders address, receiver address, and coins. A block also has a hash. The hash of the block is always unique. Once a block is created, its hash is being calculated. Changing something inside the block will cause the hash to change. The third element in the block is the hash of the previous block. It creates a chain of blocks that makes the blockchain secured from tampering [20].

**Figure 5:**
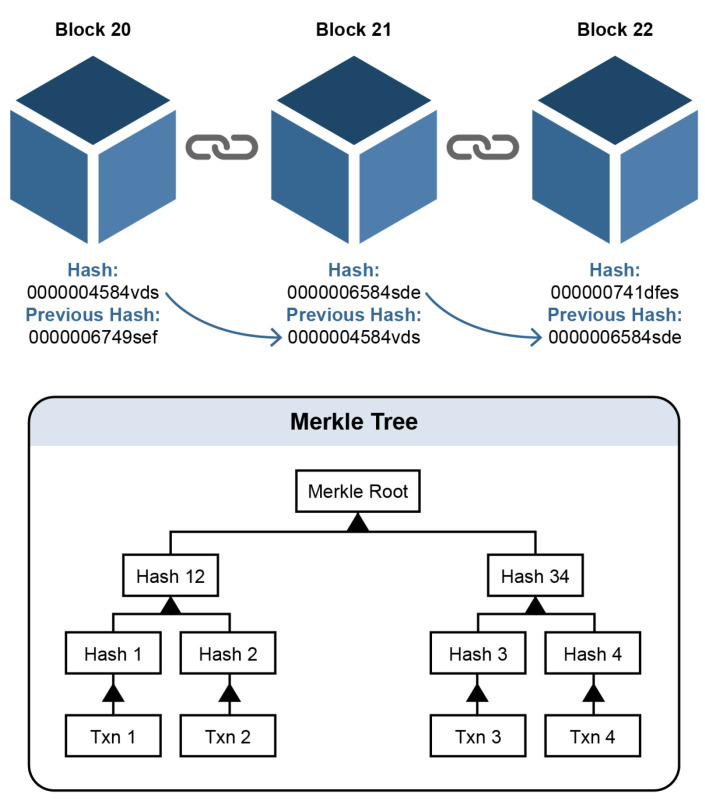
Blocks forming blockchain using hash signature.

In Figure 5, we have a chain of three blocks as we can see that each block has a hash and hash of the previous block. So the block number 21 contains the hash of the block number 20 and block number 22 contains the hash of the block number 21. If we tampered with any block, the hash of the block would be changed. It will become invalid, and the following blocks will also become invalid as they no longer hold the previous block’s hash.

Blockchain is not going to replace existing business logic. It is more of a log-based system, recording what has happened, as it is happening, rather than being the source of what to do with money from a customer, etc. It is a linked list type log data that is read and writeable by many participants. Blockchain technology passed through three eras. The blockchain 1.0 era passed through Bitcoin, the blockchain 2.0 era was marked by smart contracts, and the blockchain 3.0 era for applications in the social field [21]. Blockchains are classified into three categories. They are public, private, and permissioned. Bitcoin is a classic example of a public blockchain, in which any participant can join, read, and write data without requiring permission from a central authority. The private blockchain is restricted to members of an organization who are recognized and trustworthy. Permissioned blockchains are an example of a collection of businesses or consortiums where members are required to sign a legal contract in order to obtain access to, read, and write the blockchain. A summary of this classification is summarized in Table 1. Mohammad Dabbagh et al. published a barometric study on the evolution of blockchain. It describes the distribution of blockchain publications, most investigated research areas, the most influential papers, and the prominent funding agencies for research on blockchain [22]. Jameela AL-Jaroodi and Nader Mohamed surveyed the acceptance of blockchain in the industry. This survey shows that a wide range of industrial domains is starting to adopt or consider adopting blockchain to facilitate their operations to streamlining processes, enhancing security and data sharing, increasing efficiency, and ultimately reducing costs to gain a competitive advantage [23].

### 3.1. Basics of Blockchain Technology

This section will introduce the basic concepts that need to understand blockchain technology.

#### 3.1.1. Cryptography

Cryptography is the process of using Encryption and Decryption techniques in communications. Encryption is the process of converting our message or information into some coded message that cannot be understood by unauthorized parties. It is the core of the blockchain technology.

#### 3.1.2. Cryptographic Hash Function

**Figure 6:**
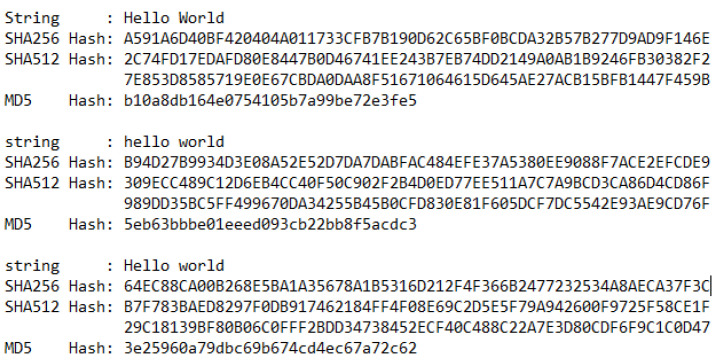
Hash values generated by different Hashing Algorithms.

A cryptographic hash function is a mathematical algorithm that maps data of arbitrary size to a bit array of a fixed size. SHA256 is an algorithm developed by the National Security Agency of the USA. SHA256 and MD5 algorithms takes the data input and processes it through a sequence of complex mathematical calculations and other transformations [24]. For computers, the data are just the binaries 1 and 0. This process outputs a fixed string of characters called a hash value or digest of the data. The hashing process is designed so that even the tiniest change in the document will result in a completely different hash. It is not encrypted because the SHA256 algorithm does not encode the information, and the hash cannot be reversed into the original data. The primary purpose of hash is a comparison, not the encryption. Figure 6 is showing the hash value of different strings generated by some famous hashing algorithms.

#### 3.1.3. Symmetric abd Asymmetric Encryption

In Symmetric Encryption, a single passphrase (key) is used to encrypt and decrypt the message. For example, Alice wants to send a classified document to Bob. She uses a key to encrypt the document and send it to Bob. Bob cannot read this document as he does not have the passphrase or key to decrypt the document. Alice needs to send this passphrase to Bob, but she cannot send it securely over the email, etc. as the attacker can attack it. Therefore, she has to find a secure way to handover this key to Bob.

**Figure 7:**
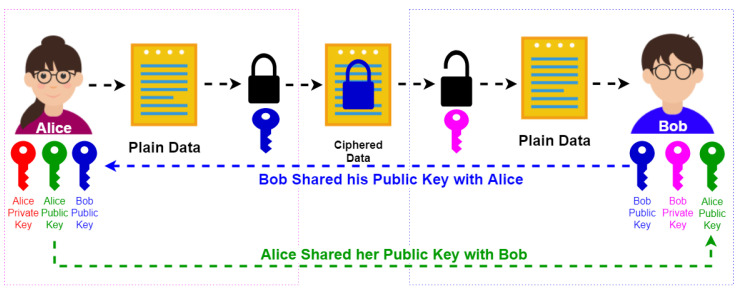
The Asymmetric Encryption Process.

In Asymmetric Encryption, both Alice and Bob generate a keypair (public key + private key) on their machines [25]. The RSA Algorithm generates a public and private key pair that is mathematically linked to each other. The public key is used to encrypt the data, and only the matching private key can decrypt that data as illustrated in Figure 7. The private key can not be derived from the public key. Bob and Alice can now exchange the public keys without any fear of each other, over the Internet or via any public means. Alice now encrypts the classified document with Bob’s public key. Alice herself now cannot decrypt that document. That document can only be decrypted with Bob’s private key.

#### 3.1.4. Digital Signature and Digital Certificate

Bob wants to send a document to Alice by electronic means. There is nothing secret about the document. Alice wants to make sure that Bob sends it, and nobody else made any changes to it on the way. Before sending the document to Alice, Bob will digitally sign this document [26]. Bob’s machine program will create SHA256 of the document and then encrypt that hash with Bob’s private key. The resulted output is called a digital signature [27]. The algorithm will embed this digital signature into the document before sending it to Alice. On receiving the document, Alice’s machine will extract the digital signature from the document and decrypt it with Bob’s public key. If the decryption is successful, that means Bob signs the document. Now the machine will calculate the hash of the document, excluding the digital signature. If the calculated hash matches with the decrypted hash, the document is original and intact. Remember that Bob and Alice do not care if someone else reads the document.

**Figure 8:**
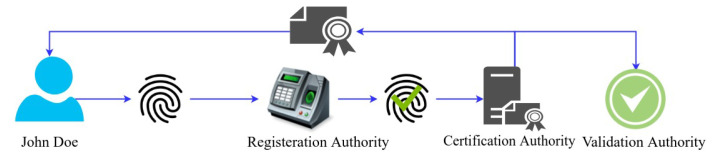
Digital Certification Authority Issues Digital Certificate After Verification of the User.

A hacker may be pretending to be Bob. He could generate a pair of public/private keys and a fake document, hash it with SHA256 and digitally sign it. That is where a digital certificate comes in handy. Bob can apply for a digital certificate from a well-known, and well-trusted organization called a certification authority. Bob will send their public key and other information to the certification authority as part of this process. The certification authority carefully checks that Bob is the one he pretends to be in the data. They then send them a particular type of file called a digital certificate [28]. It contains details about Bob alongside the information about the certification authority. This certificate also has an expiry date and Bob’s public key. Now Bob can send the digital certificate with the document to Alice. Alice can inspect the certificate first, and then after she trusts that the certificate is about Bob, she can use the public key extracted from the certificate to check the digital signature of the document. The certification authority is watching Bob carefully. Anything the certification authority sends to Bob is signed with their digital certificate provided by the higher certification authority as illustrated in Figure 8. In 2000, a law was passed in the UK called the electronic communication act. This law makes the digital signature legally binding, and this allowed businesses to thrive on the web [29,30].

#### 3.1.5. Proof of Work

The proof of work concept was initially introduced in 1993 to prevent denial of service attacks and spam on the network by requiring some work from the service user [31]. It requires some minimum processing time by the computer. Bitcoin introduced an innovative way of proof of work called consensus algorithm. It needs to solve a complex mathematical puzzle. For example, the hash value of a block must start with 13 zero, which is also called its difficulty. A particular set of nodes called miners put a string of numbers at the end of the block data and calculate its hash until they meet the requirements. The number which fulfills this requirement is called the nonce. The probability of such a number is about 1 in a billion digits. Therefore, the only way to find such a number is guess and check. The current time of creating a bitcoin block is about 10 min.

#### 3.1.6. Peer-to-Peer Network

A network is a group of interconnected communication devices connected by the wire or wireless media by switching or over the Internet. The one model is a client-server model. The server is held by a single company that performs all the tasks requested over the network. A central database is an example where a central authority stores all the data. To transfer one device’s data to other devices, it first goes to the central server and then is distributed to the other devices. In a peer-to-peer network, the devices are directly connected with each other [32]. The connected devices act as server and client simultaneously. In this kind of network, all computer devices are working together to share data with each other. The blockchain is a peer-to-peer network of devices. There is no central storage in the blockchain network, and all the connected devices have an identical copy of the data passes to each other, including network information. The devices on the blockchain network are called nodes or peers.

#### 3.1.7. Smart Contracts

Smart contracts are prevalent at present. Nick Szabo first used the term smart contract in 1997 before bitcoin was created. Smart contracts are similar to contracts in the real world. The only difference is that they are entirely digital. In fact, a smart contract is a small computer program that is stored inside the blockchain. The smart contracts code cannot be tampered with and distributed; everyone validates the smart contract’s output [33]. A single person can not force the smart contract code change as the output from the tampered smart contract will be identified as invalid on the blockchain.

## 4. Hyperledger Fabric

Hyperledger Fabric is a programmable blockchain network that encapsulates the business logic implementation, and application of the business network by way of smart contracts or chaincode. Some more essential components and services provided by Hyperledger Fabric, an open-source blockchain platform for enterprises, which made it a strong candidate of choice for this work, are described below.

**Figure 9:**
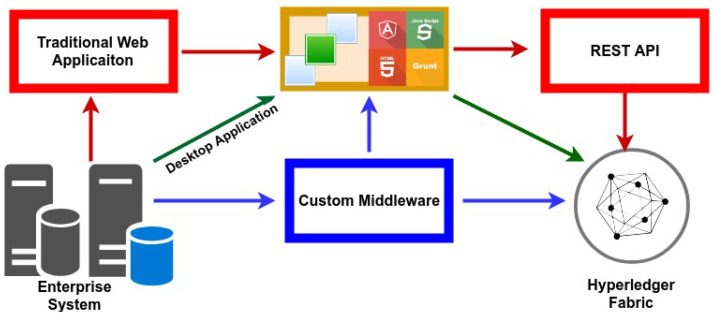
Hyperledger Fabric Application Development.

- **Permissioned Network**
  Individuals downloaded the program and instantly started transacting anonymously on a public blockchain network. This is an acceptable way to operate in corporate networks. Anonymity is not permitted on business networks. Members of a business network are continuously recognized by their unique identifiers and given responsibilities. The Hyperledger Fabric is a permission-based network that routes transactions according to established identities and responsibilities. Authentication is required for all users and components on the Hyperledger Fabric network. The Hyperledger Fabric assigns these entities their network identities through Membership Service Providers (MSP) and Certification Authorities (CA), which use Public Key Infrastructure (PKI) to approve and verify users and components.
- **Confidential Transactions**
  Confidentiality with unrelated parties is critical in a wide variety of business settings. Corporate networks often seek to keep their transactions very confidential from unrelated parties and reveal them only to the counter party. Hyperledger Fabric has a channel feature that allows transactions between defined parties to remain private. Each channel has its own ledger, and a consortium member connecting to the same network may have several channels.
- **Consensus and Policy Support**
  Members of the consortium create many policies, decisions, rules, and regulations that regulate the consortium’s functioning. Typically, consortium makes decentralized choices. Numerous administrators from member organizations vote by majority to make network modifications that affect the members of the consortium or business network. This kind of decentralized decision-making system necessitates the establishment of governance and decision-making structures. Hyperledger Fabric technology supports decentralized administration by way of policies.
- **Identity Management**
  PKI (Public Key Infrastructure) is used by Hyperledger Fabric to manage identities. It includes two tools for identity management: Active Directory integration and Fabric-CA Server. Figure 8 illustrates a common method for creating IDs. The identity owner provides the registration authority with evidence of identification. The registration authority verified and sent the user’s details to the certification authority. The certification authority generates an x509 certificate and returns it to the owner and validation authority for validation purposes. The identity is also required for the other components, such as peers and orderer nodes, to participate in the network.
- **Efficient Processing**
  To allow the network to operate in a concurrent and parallelism-friendly manner, transaction execution is kept independent from transaction ordering and commitment. Nodes in the network are assigned a role that corresponds to one of these activities, which is determined by the kind of node in the network. As a result of this concurrent execution, the processing efficiency of each peer is increased, resulting in faster transaction delivery.
- **Fabric Channel**
  Essentially, a Hyperledger Fabric channel is a private subnet of communication that is established between two or more particular network users for the aim of facilitating private and confidential transactions. Members (organizations), the shared ledger, chaincode apps, and the ordering service node all contribute to the definition of a channel (s). In the network, every transaction is carried out via a channel in which all parties are required to be verified and allowed to transact on that channel. Each peer that enters a channel has its own identity, which is provided by a Membership Service Provider (MSP), which authenticates each peer in relation to the other peers and services in the channel.
- **Endorsement**
  Every chaincode is connected with an endorsement policy, which applies to all of the smarts contracts that are specified inside that chaincode. This policy specifies which organizations in a blockchain network are required to sign a transaction produced by a certain smart contract in order for that transaction to be deemed legitimate by the network.
- **Valid Transaction**
  During the validation process of a transaction that is disseminated to all peer nodes on the network, there are two stages. First and foremost, the transaction is verified to verify that it has been signed by the organizations that have been stated in accordance with the endorsement policy. Second, it is verified that the current value of the world state corresponds to the read set of the transaction that was signed by the endorsing peer nodes at the time of the transaction signing. If a transaction satisfies both of these criteria, it is considered to be legitimate. Transactions are added to the blockchain history in both legitimate and invalid ways, but only valid transactions result in a change to the global state.
- **Ordering Service**
  When it comes to Hyperledger Fabric networks, there is another kind of node known as an orderer, which is in charge of overseeing “transaction ordering” and constructing the final block of transactions before they are transmitted to be committed in each peer’s ledger. The Ordering Service is made up of a collection of orderer nodes, which are connected together. It is the Ordering Service nodes’ responsibility to accept transactions from numerous applications after the endorsement flow has happened, and to organize batches of these transactions into a well-defined order and bundle them into the final block of transactions. In addition to their ordering responsibilities, the orderers are in responsibility of maintaining the “consortium” list, which is comprised of a list of organizations that have the ability to establish channels and are in charge of enforcing the most basic access control for the channels.
- **Network Authentication**
  Peers, orderers, client apps, and administrators are some of the various entities that may be found in a blockchain network. X.509 digital certificates are required for each of these organizations in order for them to be identified digitally. These identities are a critical component of the authentication process since they define the precise rights over resources and access to information that entities have in the network, making them a very essential component.
- **Application Development and Integration**
  Integrating the Hyperledger Fabric blockchain with an existing business system is very important to encourage the organizations adopt blockchain technology. Each company in the network may customize the interaction system to meet its own requirements. Fabric front end applications can be developed independently using RESTful APIs as middle-ware, or the custom middleware can also be designed using one of the SDKs provided by Hyperledger Fabric (Figure 9).

### 4.1. Transactions Workflow in Hyperledger Fabric

The transaction process in a Hyperledger Fabric network may be split into three major stages, which are described below.

1. **Proposal**
  When a client application requests approval from the necessary group of peers, this is referred to as the first step of the transaction approval process. On receiving a transaction proposal, each of these peers (who are acting in the role of endorsing peers) executes a chaincode to produce a transaction proposal response, after verifying and signing the incoming transaction proposal. The first step is completed after the application has obtained a sufficient number of signed proposals (depending on the requirement stated in the endorsement policy), and the second phase is completed once the application has received a sufficient number of signed proposals.
2. **Ordering Transactions**
  Transactions comprising approved transaction proposal replies are sent to an ordering service node by application clients at this step of the process. In subsequent blocks, the ordering service nodes collaborate to collectively establish a well-defined sequence, which is then packaged into the next block that is added to the blockchain. This step concludes with the blocks being stored to the orderers’ personal ledgers and then broadcasting the block to all of the peers who have connected to the orderers’ ledger.
3. **Validation and Commit**
  It is at this phase that the orderers distribute the blocks to all of the peers who are linked to them. While each peer will process this block separately, they will do it in precisely the same manner as every other peer on the channel, ensuring that the ledger remains consistent. After that, the peers will verify that the transaction has been endorsed and that it conforms with the endorsement policy in place (such as the application has done in Phase 1). After verifying the endorsement of the transaction and that the current state of the ledger is compliant with the state when the proposed change was produced, it commits and updates its copies of the ledger before committing and updating its copies of the ledger. Finally, the block events produced by the peers bring this phase to a close, events to which the application may listen in order to be notified when a transaction is committed to the ledger.

### 4.2. Rationale of Choice

An organization’s choice on which technology to use is a long-term investment. One of the most significant advantages of using an open source business application is the great degree of flexibility that is allowed by open source code, modular components, and standard compliance, among other things. This allows a business to quickly and readily change technology in order to achieve real user-friendliness. For example, if a company currently employs Kafka clusters as a message protocol in their system, we may use it as a consensus mechanism in the Hyperleger Fabric blockchain network in place of the RAFT orderering service, saving both time and money. Furthermore, we may use other existing consensus algorithms or build our own light weight consensus algorithm for IoT-platforms in order to overcome the processing power limitations of the platform. No one vendor needs to be used by enterprise and corporate networks; instead, they may choose from among the most innovative and active communities, taking advantage of the fast pace of innovation in the blockchain technology industry.

## 5. Blockchain-Based Approach for Strategic Grain Reserves Management

The grain reserves management is a multi-link sector. It includes exploration, procurement, processing, storage, and reserves release. A large number of reconciliations between different departments and track of the work and transactions require. Moreover, it is incredibly flexible and cost-efficient as it does not require some particular infrastructure and costly servers. We have developed cost-effective solutions for developing countries where finance is a big hurdle in accepting modern technology. The following subsections briefly describe working of our proposed architecture.

### 5.1. Business Consortium

**Figure 10:**
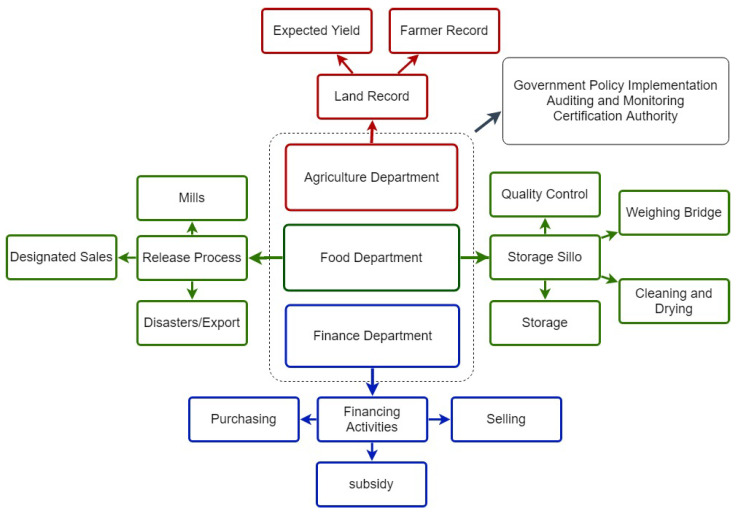
An overview of the proposed consortium.

Hyperledger Fabric is a consortium-oriented blockchain platform. Figure 10 shows the consortium configuration of our proposed system. The consortium consists of an agriculture department, food department, and finance department. The agriculture department has the record of the agricultural land and the farmer currently farming on that piece of land. They also maintain a record of the current crop on a particular land and expected yield too. The department is also responsible for purchasing, storing, and release of the grains to the mills and food product producers on a quota basis. The finance department is responsible for providing finance and an overview of the payments. The grain elevator operator determines the grain’s grade and quality and purchases the grain from the farmer. Some critical factors to be considered while storing the grain are temperature, moisture, and storage duration. The consortium makes policies to conduct operations and updates its planning and procedure to carry on all the operations. To ensure the secure tracking of all the operations using Hyperledger Fabric blockchain and chaincode the agriculture department update all the record of the agriculture land, current crop, and farmer on the system. The agriculture department also needs to update the crops’ growth and expected yield as it conducts the survey. The food department purchases the crop from the farmer by financing from the finance department. The crop samples are brought to the site, where they are passed or rejected by the quality control lab. The crop is then brought to the center where it passes through the weighing bridge cleaning and drying and then stored in the storage silo. IoT sensors and GPS systems can monitor the conditions of the silo in real time. The department is also responsible for releasing the crop to the mills or other events.

The auditing and monitoring department is receiving the live update and situation at all the stages, transaction by transaction. The audit can be conducted at any time while we have an entire tamper-proof transactions record of all the facilities. The Hyperledger Fabric blockchain uses Smart Contracts called Chaincode. The chaincode guarantees that regulations are transparent to all parties, and appropriate criteria should prompt interventions in food reserves. Chain codes are the functions in a blockchain network that accept input in the form of transactions and deliver alerts to the network’s members to monitor and detect any rule violations. The Hyperledger Fabric is an entirely permissioned network. Each component of the system, including users and the operator, must have its own identity. So no attempt of violation can be hidden in any way.

### 5.2. Application Server

**Figure 11:**
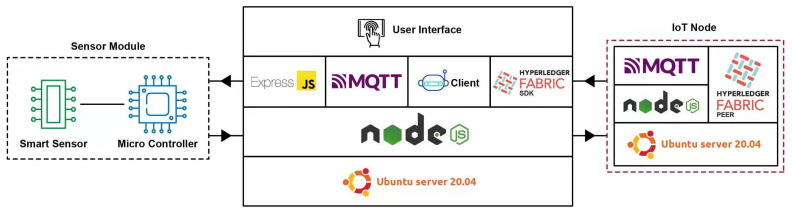
Application Node Architecture.

Figure 11 is demonstrating the general application server components. The application server is based on NodeJS, which hosts Fabric SDK, Express.js, MQTT.js, and SQL/Mongoose. Express.js provides RESTful APIs and various functions to send, receive, and access data from ERP infrastructure at the organizations. This application server provides a generic interface that can generate a new user interface or integrate it into an already existing application interface. Every current organization or future organization joining this venture can integrate this solution into their existing system according to their needs with very minimum effort due to its modular nature and simplicity.

### 5.3. Blockchain Network Architecture

**Figure 12:**
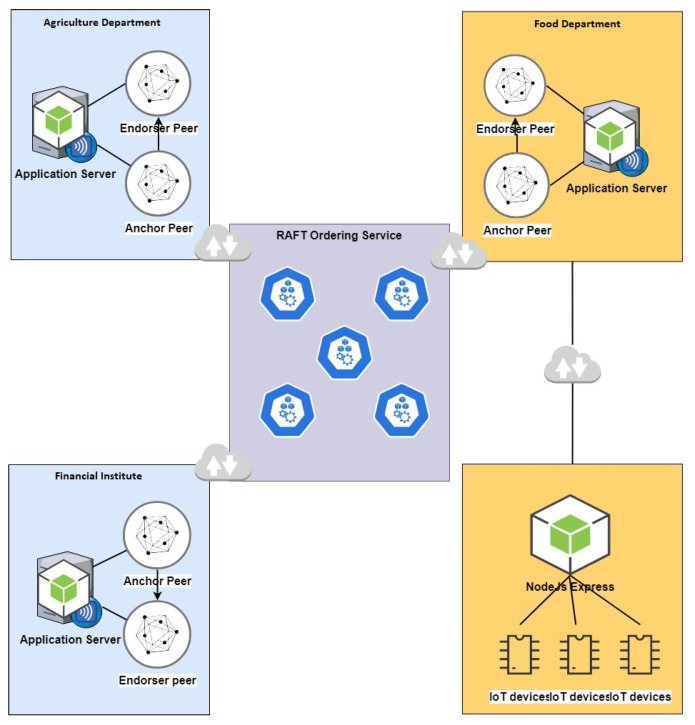
Hyperledger Fabric Blockchain Network.

Figure 12 is an illustration of a blockchain network diagram. The peer is a bridge between the fabric network and the real world. Organizations are connected with their peers to obtain access to the network. It is also a place where the ledger is stored. Remember that it is distributed to all of the peers connected to a particular channel. Every channel has one single ledger, and every participant has an exact copy of the ledger. The ledger is logically stored on the channel, but it is stored on the connected peer nodes. We still need smart contracts to interact with the ledger.

The Hyperledger Fabric blockchain uses Smart Contracts called Chain Code. The chain code can ensure the transparency of the rules for everyone, and relevant criteria should trigger food reserves interventions. In a blockchain network, the chain codes are the functions that take input in transactions and sent notifications to the network’s participants to monitor and notice any violation of the rules. The Hyperledger Fabric is a fully permission-ed network. Each component of the system requires carrying its own identity, including users and operators. So no attempt of violation can be hidden in any way. The chaincode is deployed on all the peer nodes. The chaincode is not connected to the channel, but it is hosted on the peer nodes. Now our network is ready where all of the three organizations have an equal right on the network.

### 5.4. Internet of Things Integration

The Internet of Things is a broad term that refers to a network of all these sensors capable of collecting data from their surroundings and feeding it to algorithms running on processing nodes located anywhere in the world through the Internet or a local area network. Data has surpassed oil as the most valuable resource. Industry 4.0 and agriculture 4.0 are built on the Internet of Things. The growing usage of intelligent technologies such as machine learning, cyber-physical systems, and the Internet of Things is changing human existence into entirely reliant on data generated by surrounding objects and information production. The world’s technical advances are now directly proportionate to global automation, facilitated by the Internet of Things and specific other physical systems. While the Internet of Things manages connectivity between items and machines, cyber-physical systems are machines that control or monitor a mechanism by computer-based algorithms. These linked objects will communicate with one another and make their own choices. The algorithms are fed data from a variety of sources, including smart sensor devices.

**Figure 13:**
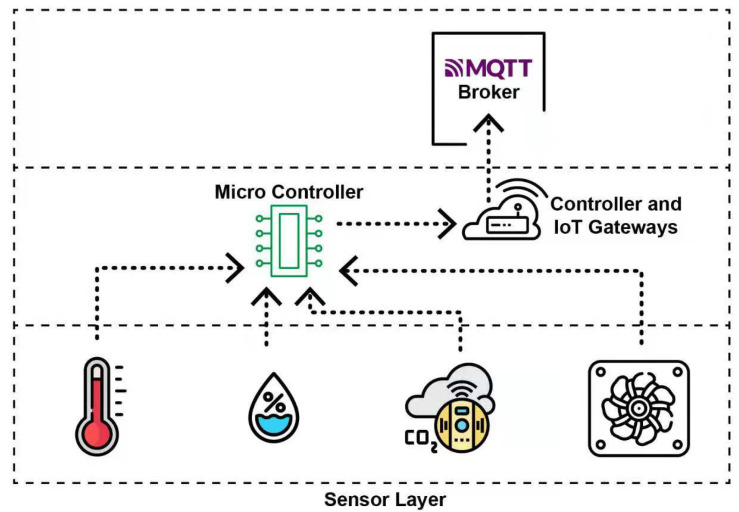
IoT Layer Configuration.

A large number of food grains are wasted due to improper temperature and humidity at storage facilities. The grains become infested with mold and insects due to the lack of environmental monitoring techniques. When the grains go through the drying process, temperature and moisture present in the grain can cause stress, cracks on the grain kernel. The IIoT [34] focuses on machines, sensors, and devices to provide various services such as tracking objects [35], environmental monitoring [36], healthcare industry [37], traffic management [38], smart cities [39], and many more. International Society of Automation (ISA) provides various standards for implementing automation in the industry and so on [40]. Figure 13 is self describing the IoT system implementation.

## 6. Qualitative Analysis of the Proposed Blockchain Solution

Many existing technological options are available for traceability and control. However, blockchain technology provides another level of trust by a tamper-proof shared ledger. The transparency it gives and its tamper-evident nature creates trust in low-cost IT solutions. It stores every transaction in the network, and all the parties in the system have the same data. It maintains an undeniable record of what is added to the data, by whom, in the chronological order. The data cannot be removed in the blockchain. By enabling each party to see the same data, in near real-time, blockchain can help eliminate complicated and costly data reconciliation required by most systems in the world today.

### 6.1. Decentralized Database

Blockchain is a decentralized platform for conducting transactions. In traditional databases, we have a centralized authority to store all the data. The conventional client-server model is a perfect example of this. The conventional model is an easy task for hackers or criminals as all the data is stored in one place, and the worst scenario is if the centralized entity became corrupt. The main idea behind blockchain is if we want to make a transaction with anyone, and we can do it directly without any third party, and both of us have a tamperproof record of the transaction. All the nodes maintain the data on the blockchain system, and no node has full access to it.

### 6.2. Persistency

The data on the blockchain is persistent as these data are distributed along with the network. Each node has control of its data. All the transactions are transparent and tamperproof, which makes the complete blockchain persistence.

### 6.3. Validity

The transactions put on blockchain may be validated by some of the other nodes in the network. Any mistakes as malicious transactions can be detected very quickly. The system consists of three primary roles. (1) propose a transaction (2) validates which transactions to accept (3) Lerner who accept the chase value.

### 6.4. Anonymity and Identity

The public blockchains such as bitcoin and Ethereum provide complete anonymity. A single user can obtain multiple identities. The public blockchain has not any central entity to maintain the identities. The private blockchains, on the other hand, required the identity to access the blockchain network. In permission-ed blockchains, the identities are provided by the consortium which is operating the blockchain under consortium policies.

### 6.5. Audibility

All the blockchain transactions are time-stamped and tightly linked with the previous transaction in the chain protected by the cryptographic hash functions. Therefore, it is easy for a node to trace the history of the transaction. The blockchain’s audibility also depends on the type of blockchain, such as the public blockchain, which is fully accessible, and any node can fully trace any transaction. On the private and permission-ed blockchain, the parties’ agreements may not allow it to become fully audible for everyone.

### 6.6. Closeness and Openness

The open blockchain consists of public nodes for consensus and maintenance of transactions. Anyone can join these open blockchains and start transactions or can become part of the consensus process. The private and permission-ed blockchains are closed or semi-opened. There are preselected nodes for the consense on the data. These blockchains rely on a single entity called a consortium for the permissions.

### 6.7. Traceability and Audit

In our proposed blockchain-based system, all the information regarding expected yield, finance, available stock and conditions at silo are available to all stakeholders in real time. Beginning with the land record and expected yield the policy makers can make their policy with real time data availability in minimum time. The crop sold by a farmer cannot be greater than a set percentage of expected yield and have a complete record of the land and farmer. In addition, the grains of different qualities cannot be mixed and total volume of purchase and sale is known by all the stakeholders in real time. It is possible that some stakeholder entered the wrong and fraudulent information into the system. If that data is caught at some stage, all the stakeholders know with 100% confidence who originated that data. Continuous monitoring of the silo location and quality control with traceable identifiers per lot and the realtime transaction traceability and availability to all the stakeholders makes it more corruption secure.

## 7. Conclusions

We proposed a blockchain-based solution to manage the strategic grain reserves of a country in this article. We presented possible general motivations to apply blockchain technology in food reserves management. We described the very generic detail of the blockchain systems overview and how our system can be applied applied to provide tamper-proof data availability. Moreover, many governments are also showing an interest in using blockchain technology in governance. Future needs include the development of a system where the different blockchains can transfer the data to each other or can be merged. Future work includes the technological demonstration of the project with the Artificial Intelligence for data analytic and data visualization.

## Figures and Tables

**Table 1:** Classification of Blockchains.

	Public Blockchain	Private Blockchain	Permission-Ed Blockchain
**Read Access**	Not Required	Within the Organization	Controlled
**Write Access**	Not Required	Within the Organization	Controlled
**Consensus Process**	Open Access	Within Organization	Within Consortium
**Scalability**	High	Low	Medium

## Data Availability

Data sharing is not applicable for this article.
